# Biogeography of curimatid fishes reveals multiple lowland–upland river transitions and differential diversification in the Neotropics (Teleostei, Curimatidae)

**DOI:** 10.1002/ece3.8251

**Published:** 2021-11-09

**Authors:** Bruno F. Melo, James S. Albert, Fernando C. P. Dagosta, Victor A. Tagliacollo

**Affiliations:** ^1^ Department of Ichthyology American Museum of Natural History New York New York USA; ^2^ Department of Biology University of Louisiana at Lafayette Lafayette Louisiana USA; ^3^ Faculdade de Ciências Biológicas e Ambientais Universidade Federal da Grande Dourados Dourados Brazil; ^4^ Museu de Zoologia Universidade de São Paulo São Paulo Brazil; ^5^ Instituto de Biologia Universidade Federal de Uberlândia Uberlândia Brazil

**Keywords:** Amazonia, Andes, Brazilian Shield, diversification, GeoHiSSE, Guiana Shield, Neotropics, river capture

## Abstract

The Neotropics harbors a megadiverse ichthyofauna comprising over 6300 species with approximately 80% in just three taxonomic orders within the clade Characiphysi. This highly diverse group has evolved in tropical South America over tens to hundreds of millions of years influenced mostly by re‐arrangements of river drainages in lowland and upland systems. In this study, we investigate patterns of spatial diversification in Neotropical freshwater fishes in the family Curimatidae, a species‐rich clade of the order Characiformes. Specifically, we examined ancestral areas, dispersal events, and shifts in species richness using spatially explicit biogeographic and macroevolutionary models to determine whether lowlands–uplands serve as museums or cradles of diversification for curimatids. We used fossil information to estimate divergence times in BEAST, multiple time‐stratified models of geographic range evolution in BioGeoBEARS, and alternative models of geographic state‐dependent speciation and extinction in GeoHiSSE. Our results suggest that the most recent common ancestor of curimatids originated in the Late Cretaceous likely in lowland paleodrainages of northwestern South America. Dispersals from lowland to upland river basins of the Brazilian and Guiana shields occurred repeatedly across independently evolving lineages in the Cenozoic. Colonization of upland drainages was often coupled with increased rates of net diversification in species‐rich genera such as *Cyphocharax* and *Steindachnerina*. Our findings demonstrate that colonization of novel aquatic environments at higher elevations is associated with an increased rate of diversification, although this pattern is clade‐dependent and driven mostly by allopatric speciation. Curimatids reinforce an emerging perspective that Amazonian lowlands act as a museum by accumulating species along time, whereas the transitions to uplands stimulate higher net diversification rates and lineage diversification.

## Graphical abstract

The time‐calibrated molecular phylogeny and geographic range evolution of the Neotropical family Curimatidae show a Late Cretaceous origin in lowland basins of South America with subsequent colonization to upland basins at multiple times during the Cenozoic. Dispersal events, especially in the species‐rich genera *Cyphocharax* and *Steindachnerina*, were prevalent in the Brazilian Shield during the Paleogene and Neogene. Colonization of uplands increased net diversification rates in some curimatid subclades.

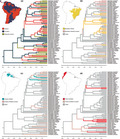

## 1 INTRODUCTION

Neotropical freshwater fishes (NFF) constitute the most diverse continental vertebrate fauna on Earth, with over 6300 valid species, approximately 80% within Characiphysi (Characiformes, Gymnotiformes, and Siluriformes), exhibiting a bewildering range of morphological, behavioral, and ecological phenotypes (Albert & Reis, 2011; Albert et al., 2020; Reis et al., 2016). Among the most consequential geological and climatic events driving species diversification in the Neotropics are: (1) Early/Late Cretaceous (*ca*. 120–95 Ma) fragmentation of western Gondwana and Paleogene (*ca*. 66–22 Ma) rise of the Central and Southern Andean cordilleras (Granot & Dyment, 2015; Lundberg, 1993), (2) Neogene (*ca*. 23.0–2.6 Ma) and Quaternary (*ca*. 2.6–0 Ma) uplift of the northern Andes and paleogeographic transformation of the Caribbean‐draining Proto–Orinoco–Amazonas (POA) basin to the modern Amazon and Orinoco basins (Albert et al., 2018; Hoorn et al., 2010), (3) Plio–Pleistocene (*ca*. 5.3–0.01 Ma) global climate oscillations and eustatic sea‐level changes driving marine transgressions and regressions (Abreu et al., 2019; Bloom & Lovejoy, 2011; Thomaz & Knowles, 2018; Thomaz et al., 2015), and (4) perennial river captures (*i.e*., geomorphological diversion of drainages to adjacent river systems) among tributaries of the large lowland river basins (Roxo et al., 2014; Ruokolainen et al., 2019; Santos et al., 2021; Tagliacollo et al., 2015). Paleogeographic reconstructions from previous studies have shown how these geological and climatic events shaped South American landscapes and biodiversity through time (Lundberg, 1993; Lundberg et al., 1998; Hoorn et al., 2010; Albert & Reis, 2011; Albert, Val, et al., 2018; Tagliacollo et al., 2015; Tagliacollo et al., 2017 Albert et al., 2020; Fontenelle et al., 2021).

As with all continental biotas, variation in both climate and landscapes affects the formation of NFF assemblages of lowland and upland river basins. Lowlands are defined as low‐elevation (<250–300 m) wetlands, floodplains, lakes, and large river channels in the Amazon, Orinoco, and Paraguay basins, including large tributaries such as Madeira, Negro, Purus, Putumayo, Solimões, and Ucayali rivers. Uplands are defined as higher elevation (>250–300 m) basins associated with the Andean cordilleras (*e.g*., Magdalena‐Cauca, Upper Meta) or cratonic highlands covering most of central and southeastern Brazil (the Brazilian Shield; *e.g*., Paraná, São Francisco, Upper Tapajós, Araguaia‐Tocantins, Upper Xingu rivers) and portions of Venezuela and the Guianas (the Guiana Shield; *e.g*., Branco, Corantijn, Essequibo, Marowijne, and Oyapock rivers) (Albert et al., 2018; Albert & Reis, 2011). These elevation zones exhibit distinct geomorphological and environmental profiles and contrasting biogeographic patterns. Compared with upland basins, lowland basins are more centrally located on the continental platform, have larger rivers and floodplains with a greater total volume of aquatic habitat, and flow across more erodible alluvial substrates with more rapidly changing river courses. Lowland basins of northern South America (*e.g*., Amazon and Orinoco basins) also exhibit much higher species densities (*i.e*., local alpha diversity) and lower percent species endemism (*i.e*., geographic beta diversity) than do upland basins of the continental periphery (Albert et al., 2011, 2020).

The evolutionary origins of the remarkable NFF diversity remain incompletely understood, with several hypotheses linking mechanisms of diversification to regional geomorphological and climate histories (Albert & Reis, 2011; Albert et al., 2020; Lundberg et al., 1998). One hypothesis states that the older cratonic Brazilian and Guiana shields are more stable geologically and environmentally, and therefore represent an "area of origin" for the NFF fauna (Eigenmann & Allen, 1942; Albert & Carvalho, 2011: fig. 7.3). Another hypothesis states that rivers in the large lowland basins undergo faster rates of tributary exchanges due to river capture and sea‐level fluctuations, and therefore experience greater rates of both speciation and extinction than upland basins (Albert et al., 2011; Lima & Ribeiro, 2011). Thus, lowland basins have been hypothesized to generate species more rapidly, acting as evolutionary cradles of diversity, while upland basins have been hypothesized to isolate species and protect them from extinction acting more as evolutionary museums of diversity (see Albert et al., 2011; Lundberg et al., 1998).

**FIGURE 1:**
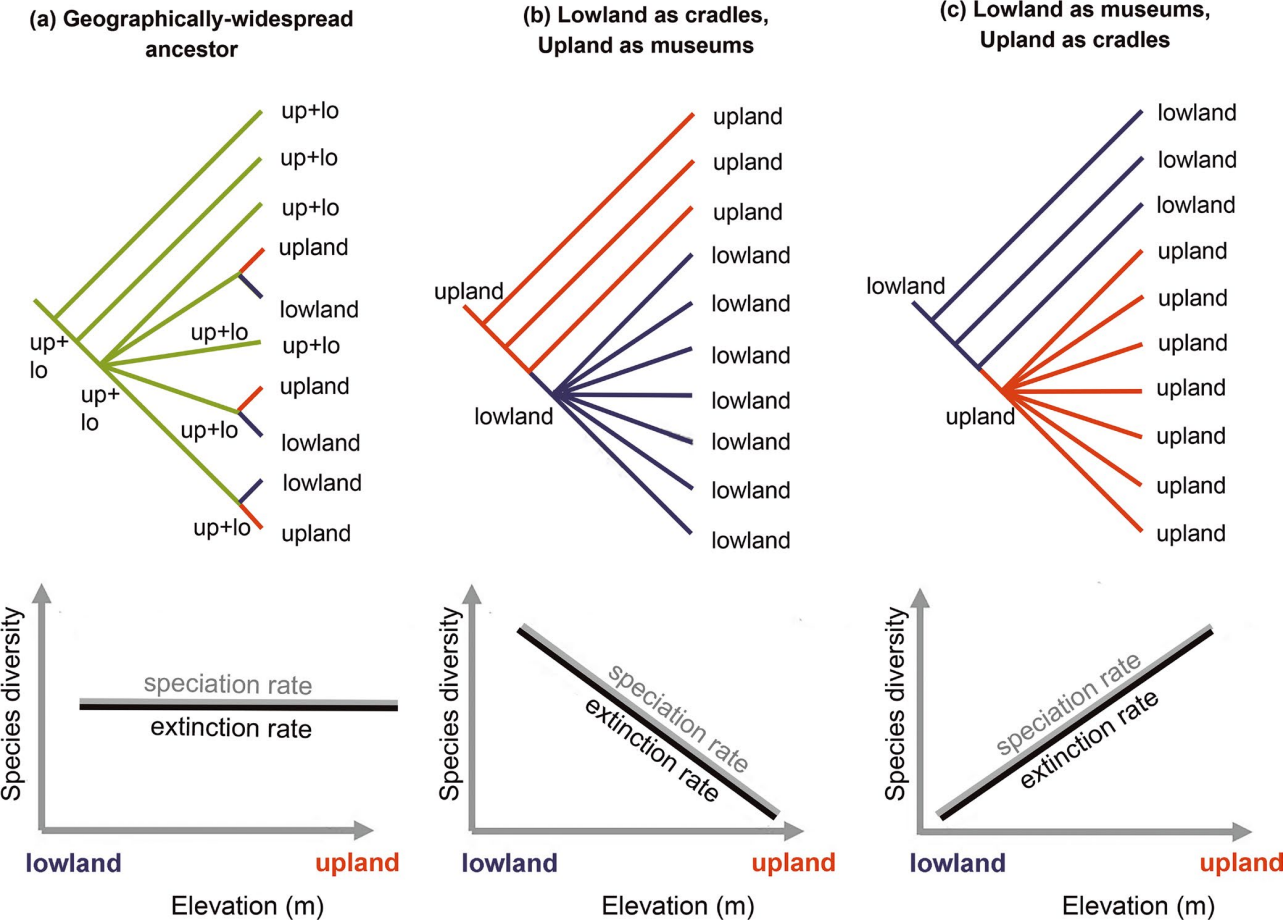
Alternative macroevolutionary models of species inhabiting lowland and upland river basins of tropical South America (from Albert et al. (2011); topologies reproduced with permission): (a) vicariance into lowland and upland descendants from geographically widespread ancestors; (b) lowlands as cradles of diversity with high diversification rates and uplands as museums with low diversification rates; and (c) lowlands as museums and uplands as cradles. Note other models are possible in which rates of speciation and extinction are decoupled (Louca & Pennell, 2020)

An evolutionary museum is a region of net species accumulation, in which regional extinction rates are lower than the combined rates of in situ speciation and immigration from adjacent regions (Stebbins, 1974). Conversely, an evolutionary cradle is a region of net species overproduction, in which regional speciation rates exceed extinction rates, resulting in an increase of species richness and biogeographic range expansion (*i.e*., dispersal) to adjacent regions (Figure 1) (Albert et al., 2011; Rangel et al., 2018; Stebbins, 1974). Although these simple macroevolutionary models do not fully summarize the complexity of most biogeographic regions, the museum‐cradle paradigm is widely used in contemporary analyses of tropical diversification (Azevedo et al., 2020; Cássia‐Silva et al., 2020; Dagallier et al., 2020; Matos‐Maraví et al., 2019; Melo et al., 2021; Meseguer et al., 2020; Rangel et al., 2018). Some caveats that apply to these models are that the taxa of a given region are not expected to be monophyletic with respect to that of adjacent regions (Figure 1) (Albert et al., 2011) and that a region may simultaneously serve as a cradle for some taxa and a museum for others (McKenna & Farrell, 2006; Moreau & Bell, 2013). Although the original museum/cradle concept of Stebbins (1974) was qualitative and descriptive, these terms are now used as shorthand for quantitative models that compare rates of net diversification among regions (*e.g*., Jablonski et al., 2006; Moreau & Bell, 2013). Although these macroevolutionary models have been tested in other tropical groups (Gaston & Blackburn, 1996; McKenna & Farrell, 2006; Moreau & Bell, 2013), none have yet been tested explicitly in the NFF fauna (see Albert et al., 2011, but see Melo et al., 2021).

Curimatid fishes, the *branquinhas*, *saguirus*, or toothless characiforms (Teleostei: Ostariophysi), are well suited to studies documenting the spatial and temporal contexts of evolutionary diversification. The Curimatidae is a broadly distributed clade of riverine‐adapted characiform fishes that inhabits both lowland and upland river basins of tropical South and lower Central America (Vari, 1988). Members of the clade are detritivorous and perform short lateral migrations (Fernandes, 1997), with some species attaining larger body [up to 32 cm standard length in *Curimata mivartii*] and population sizes, thereby constituting an important component of Amazonian riverine food webs and human fisheries (Araujo‐Lima et al., 1986; Araujo‐Lima & Ruffino, 2003; Lowe‐McConnell, 1975). Curimatids are important parts of the fish faunas in the lowland rivers of the Amazon, Orinoco, and La Plata basins, upland rivers of the Guiana and Brazilian shields (*e.g*., Essequibo, Tocantins, São Francisco, and Paraná basins), and swiftly flowing rivers west of the Andes (*e.g*., Maracaibo, Magdalena, Atrato and Pacific coastal basins from northern Peru to Costa Rica) (Frable, 2017; Vari, 1988). The alpha taxonomy and higher‐level systematics of curimatids have attracted substantial attention (*e.g*., Vari et al., 2012; Melo, 2017, 2020; Melo & Oliveira, 2017; Bortolo et al., 2018) using both morphological and molecular evidence (Dillman et al., 2016; Dorini et al., 2020; Melo et al., 2016, 2018; Vari, 1989). The family is represented by 117 valid species in eight extant genera, representing one of the ten most species‐rich families in the Amazon basin (Dagosta & de Pinna, 2019; Fricke et al., 2021; Vari, 1989).

Based on a morphological phylogeny, Vari (1988) investigated the historical biogeography of curimatids and his findings supported the delimitation of ten major bioregions in South America and the hypothesis that post‐Andean cladogenesis had moderate effect on the curimatid diversification. Vari (1988) further discussed the close relationships among fish assemblages of the Amazon, Orinoco, and Guianas, and the mixed biota for the São Francisco basin and rivers of northeastern Brazil. More recently, a molecular phylogeny refined the relationships of curimatids including *ca*. 67% of all species diversity (Melo et al., 2018), resulting in a robust evolutionary framework to reassess Vari's hypotheses and to investigate spatial macroevolutionary scenarios for curimatid fishes. Combining this phylogeny (Melo et al., 2018) with an extensive spatial database, we estimate temporal diversification and geographic range evolution of curimatids aiming to elucidate whether lowland/upland river basins act as museum or cradles of lineage diversification in the Neotropics. Our findings on curimatid fishes support an emerging perspective of Amazonian lowlands as both a cradle and a museum of diversification, accumulating species over tens of millions of years, and serving the primary source of Neotropical biodiversity (Antonelli, Ariza, et al., 2018; Antonelli, Zizka, et al., 2018), while the shield uplands act more often as a cradle of species diversification.

## 2 MATERIALS AND METHODS

### 2.1 Divergence time estimates

This study used as the phylogenetic framework a published multilocus dataset containing three mitochondrial loci, the *16S rRNA*, *cytochrome C oxidase subunit I* (*COI*), and *cytochrome B* (*Cytb*), and three nuclear coding genes, the *myosin heavy chain 6 gene* (*Myh6*), *recombination activating gene 1* (*Rag1*), and *recombination activating gene 2* (*Rag2*), with a total of 5358 characters and 151 terminals (Melo et al., 2018). The original matrix was reduced to 142 terminals by excluding nine outgroup taxa because our aim was to estimate divergence times only. Voucher information and GenBank accession numbers are available in Table S1, and the additional locality information of taxa is available in Melo et al. (2018). PartitionFinder v2.1.1 (Lanfear et al., 2012) was used to infer the optimal partitioning scheme and model of DNA evolution for partitions, in which the non‐coding *16S rRNA* was set as one partition and the remaining five coding genes were subdivided by codon position. This analysis used the greedy algorithm and the Akaike information criterion (AIC) assuming the models available in BEAST v1.8.2 (Drummond et al., 2012) ([Link], [Link]). The best maximum‐likelihood (ML) tree was obtained using the GTRGAMMA model in RAxML v8 (Stamatakis, 2014). An uncorrelated relaxed molecular clock with lognormal distribution was applied in BEAST v1.8.2 (Drummond et al., 2012), and the best ML tree was fixed as the topology with BEAST estimating only node ages.

The first calibration point was the fossil †*Cyphocharax mosesi* (Travassos & Santos, 1955) (holotype DGM 620‐P; types DGM 621‐P, MNRJ 923‐V, UNG‐854), an incomplete body skeleton from the Oligocene–Miocene boundary (*ca*. 23.8 Ma) described from the deposits of the Tremembé Formation, Taubaté basin, São Paulo, southeastern Brazil (Malabarba, 1996; Malabarba & Malabarba, 2010). This species shares with congeners modifications in the laterosensory canals of the fourth and fifth infraorbitals (Malabarba, 1996; Vari, 1989). †*Cyphocharax mosesi* has been tentatively hypothesized as close related to the extant species *C. gilbert*, *C. modestus*, and/or *C. santacatarinae* (Malabarba, 1996). The most recent molecular phylogeny of Curimatidae suggests these three extant species belong to the *C. gilbert* clade, which further includes *C. corumbae*, *C. naegelii*, *C. platanus*, *C. spilotus*, and *C. voga* (Melo et al., 2018). Thus, we used stratigraphic information of †*Cyphocharax mosesi* to constrain age estimates of the most recent common ancestor (MRCA) of *C. gilbert* clade using a lognormal prior (offset = 23.8 Ma; mean = 5.0; stdev = 7.5).

The second calibration is a constraint on the root of the tree selected to match the timing of the split between Curimatidae and Chilodontidae, as estimated from the molecular analysis of Characiformes (Melo et al., 2021). This analysis included 356 taxa and six fossil calibrations, and the time‐calibrated phylogeny revealed the split of Chilodontidae and Curimatidae at approximately 54.9 Ma (64.2–46.2 Ma, 95% highest posterior density; HPD) (Melo et al., 2021); therefore, we assigned a normally distributed prior on the root of our phylogeny (offset = 54.9 Ma; stdev = 7.5). The other available curimatid fossil †*Plesiocurimata alvarengai* (Figueiredo & Costa‐Carvalho, 1999) is controversial because of the incompleteness of informative characters preventing any phylogenetic placement of the fossil species within the family (Figueiredo & Costa‐Carvalho, 1999). The BEAST analysis was partitioned under the models of molecular evolution as determined by PartitionFinder, and a birth–death model was applied for the tree topology with branch lengths. BEAST ran 600 million generations, sampling frequencies at every 60,000th generation, which resulted in 10,001 trees. Stationarity and sufficient mixing of parameters (ESS > 200) were verified using Tracer v1.6 (Rambaut et al., 2014), and the maximum clade credibility tree was obtained from the last 9001 trees (10% burn‐in) using TreeAnnotator v1.8.2 and visualized in FigTree v1.4.3; softwares are available in the BEAST package (Drummond et al., 2012). The matrix, input files, BEAST parameters, and PartitionFinder results are available in the [Link], [Link] at Dryad (https://doi.org/10.5061/dryad.9p8cz8wfw).

### 2.2 Biogeographic regions

**FIGURE 2:**
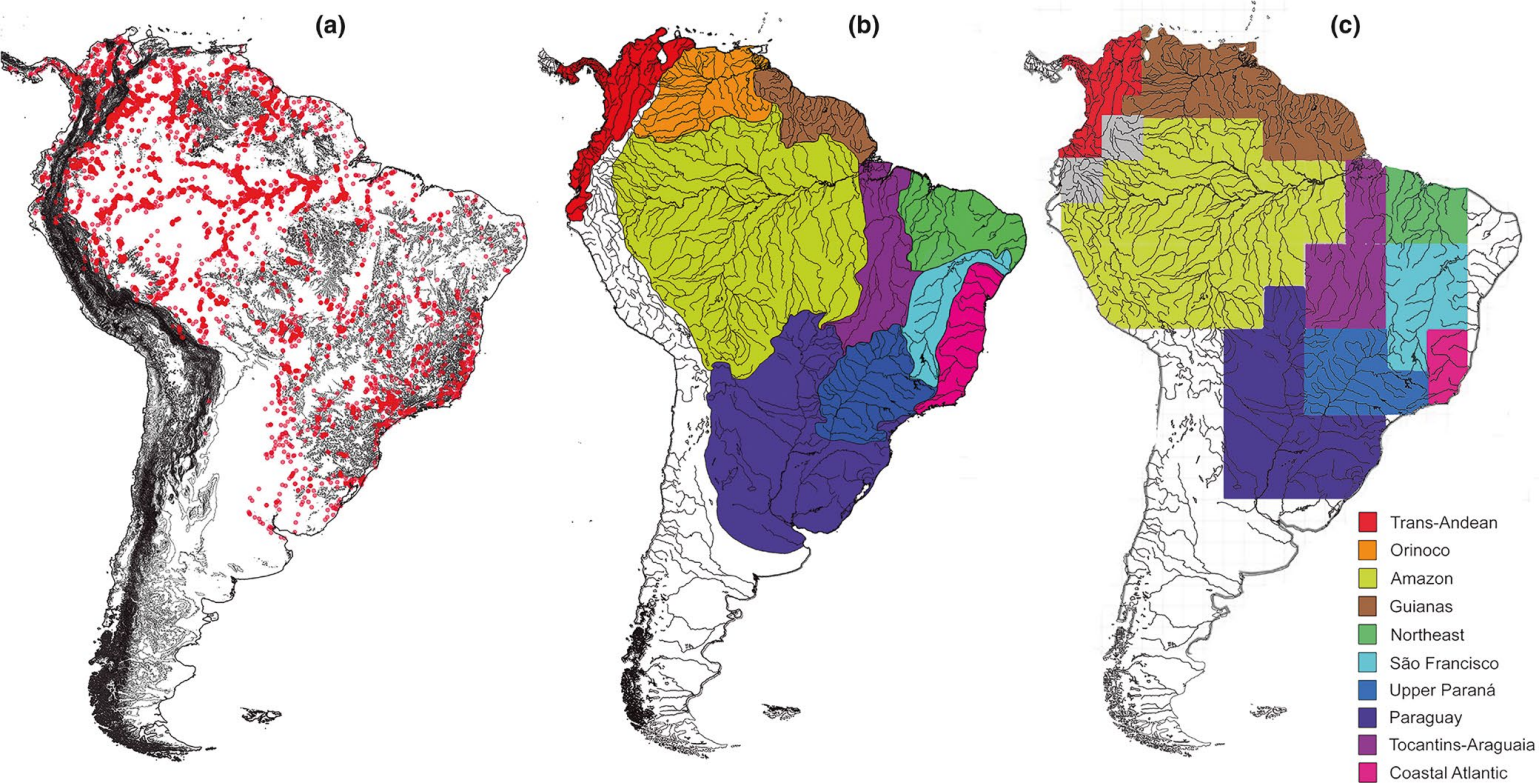
Delimitation of geographic units for biogeographic inferences of endemism predicted by bipartite network clustering. (a) Geographic coordinates for 103 species of Curimatidae across Central and South America analyzed in this study. (b) Areas of endemism for curimatid species *sensu* Vari (1988). (c) Bioregion delimitations for curimatid species resulted from Infomap Bioregions. Note similarities between the two hypotheses (b‐c), except for the connectivity between Orinoco and Guianas, the inclusion/exclusion of some trans‐Andean basins, and those of northeastern Brazil and the Paraguay basin. Number of occurrences = 5362

Vari (1988) proposed 10 regions of endemism for curimatids. We tested this hypothesis with a dataset comprising 5362 GPS coordinates from 103 curimatid species assembled from museum collections, publications, and metadata repositories such as SpeciesLink (http://www.splink.org.br). We used the R package “Biogeo” (Robertson et al., 2016) to remove duplicates and records with obvious georeferenced errors. This procedure excluded occurrences in the ocean or outside the Neotropical region, those without country names, coordinates with zero latitude or longitude, and coordinates annotated on coarse‐scale grid without decimal precision (Figure 2a). We used this assembled spatial dataset to delimit bioregions using bipartite network clustering in the Infomap Bioregions (Edler et al., 2016). This program uses an adaptive resolution of grid cells when sampling effort is uneven to generate bipartite networks (*i.e*., species vs. grid cells) clustered using the Infomap clustering algorithm. Our analysis applied the following inputs: max. cell size = 8º, min. cell size = 1º, max. cell capacity = 100, min. cell capacity = 30, and number of trials = 5.

### 2.3 Biogeographic estimates

We used the R package "BioGeoBEARS" (Matzke, 2013) to compare the likelihoods of six time‐stratified biogeographic models. The time intervals represented six epochs, from Late Cretaceous to Holocene. The epochs Pliocene, Pleistocene, and Holocene were grouped into one interval. Constraints on area connectivity (*i.e*., areas‐allowed) were similar across all six epochs, except in the last time slice where range expansions were not allowed between the cis‐ and trans‐Andean river basins. Each model incorporates a combination of three analytical procedures and the inclusion or not of the distance‐based parameter rate "x" (Van Dam & Matzke, 2016). The dispersal matrix was built using estimates of centroid points and Euclidian distances between adjacent bioregions and then rescaled by the shortest measured distance. The analytical procedures encompassed three biogeographic models: DEC model (Ree & Smith, 2008) and the probabilistic implementations of the programs DIVA (Matzke, 2014; Ronquist, 1997) and BayArea (Landis et al., 2013; Matzke, 2014). Each analytical procedure allows an exclusive cladogenetic process (DEC: subset sympatry; DIVA: widespread vicariance; and BayArea: widespread sympatry). We decided not to evaluate DEC, DIVA, and BayArea biogeographic models with the founder cladogenetic process (the "+j" parameter). Besides criticism on the parameterization of the founder events (Ree & Sanmartín, 2018), continental fish are unlikely to undergo a jump speciation process because their dispersal abilities are usually constrained by river connectivity between adjacent watersheds. None of the curimatid species included in the analyses inhabited more than five bioregions; therefore, a maximum of five combined bioregions was allowed at each node of the time‐calibrated tree. Input files of the six time‐stratified biogeographic models are available in the [Link], [Link].

### 2.4 Model selection

Estimates of AICc scores compared global likelihoods at the root node across the six time‐stratified biogeographic models (Table 1). Besides penalizing additional model parameters, AICc has corrections for small sample sizes. When sample sizes are large, AICc scores converge to standard AIC scores. The sample size was the number of terminal taxa in the time‐calibrated tree. The six biogeographic models included either two or three parameters: range expansion rate (d), range contraction rate (e), and distance‐based dispersal rate (x). The lowest AIC score indicated the best‐fitting biogeographic model. ΔAIC scores less than 2‐log likelihoods were considered equally supported (Burnham & Anderson, 2004). Two of the six time‐stratified models were considered equally supported (DIVA and DIVA + x) (Table 1). Although the two models include DIVA as their analytical procedure, they differ by the distance‐based dispersal rate parameter "x." Given that both models are statistically supported (Table 1), the simplest one with the fewer parameters (*i.e*., DIVA) was used to perform subsequent biogeographic analyses.

**TABLE 1:** Model selection among six time‐stratified biogeographic models

	Model	LnL	*n*	d	e	x	AICc	ΔAIC
M1	DEC	−276.2	2	0.02	0.01	‐	556.6	6
M2	DIVA	−273.8	2	0.02	0.01	‐	551.7	1.1
M3	BayArea	−291.7	2	0.02	0.04	‐	587.6	37
M4	DEC + x	−274.1	3	0.05	0.01	−0.779	554.5	3.9
M5	DIVA + x	−272.1	3	0.04	0.01	−0.606	550.6	0
M6	BayArea + x	−578	3	0.09	0.04	−1.043	584.3	33.7

Note

Models with ΔAIC scores less than 2‐log likelihoods are equally supported as the best‐fitting model. Each model comprised a unique combination of ML analytical procedures (DEC, DIVA, BayArea) and a distance‐based dispersal parameter rate (x).

Abbreviation: AICc, Akaike information criterion with corrections for sample sizes; d, range expansion parameter; e, range contraction; LnL, log‐likelihood; *n*, number of parameters; ΔAIC, delta AIC.

### 2.5 Biogeographic Stochastic Mapping

Biogeographic stochastic mapping (BSM) is a simulation‐based approach for estimating biogeographic scenarios conditioned on a time‐calibrated tree, biogeographic model, and its parameters (Dupin et al., 2017). Specifically, it is applied to simulate multiple equiprobable scenarios of range evolution given the best‐fitting model, therefore allowing statistical inferences of modes and counts of biogeographic events (Dupin et al., 2017). BSM scripts available in "BioGeoBEARS" (Matzke, 2013) were used to estimate modes and counts of biogeographic events in the curimatid tree. To calculate the probability of ancestral states for each node, we performed 100 BSM simulations under the simplest best‐fitting model (i.e., DIVA; Table 1). Cladogenetic events were binned in two geographic categories: sympatric (within‐basin) and vicariant (among‐basin) events. These events were further categorized by altitudinal gradients and vicariant patterns.

### 2.6 Diversification rates

We used the Geographic State‐dependent Speciation and Extinction (GeoSSE/GeoHiSSE) models (Caetano et al., 2018) in the R packages "devtools," "hisse," and "diversitree" (Beaulieu & O'Meara, 2015; FitzJohn, 2012) to estimate net diversification rates, and to test the hypothesis of range‐dependent diversification processes (*i.e*., lowland/upland). These models apply a three‐state Markov Chain allowing for species categorization in distinct (*e.g*., A or B) or combined (*e.g*., A and B) geographic units. Here, we categorized each species in a matrix with: (1) geographic range only in lowlands, (2) geographic range only in uplands, or (3) geographic range in both lowlands and uplands. Model parameters were the following: (1) speciation rate in each geographic unit (sLowands or sUplands) and in both units (sLowland and sUplands), and (2) extinction rate in each geographic unit (xLowlands or xUplands), and dispersal rates between geographic units (dLowands  = lowlands to uplands, dUplands  = uplands to lowlands). The state‐dependent models optimize orthogonal transformations of these variables such as the parameter of net turnover for the widespread range (*e.g*., 01) and extinction fractions of endemics (*e.g*., 0 or 1). There is no "extinction" parameter associated with widespread lineages (see details in Caetano et al., 2018).

The chronogram and geographic range matrix were used as the input data for testing four models—(M1) a range‐independent diversification process without hidden states (null model): This model assumes a homogeneous diversification rate across the tree independent of the ranges; (M2) a range‐dependent diversification process without hidden states (GeoSSE): This model assumes that diversification shifts are correlated to the individual ranges (uplands vs. lowlands); (M3) a range‐independent diversification process with hidden states: This model assumes shifts in diversification across the tree independent of the ranges, that is, the parameters vary among hidden states; however, they are the same within each hidden state; and (M4) range‐dependent diversification process with hidden states and multiple rate states: This model assumes shifts in diversification across the tree, and these are dependent on the geographic ranges. The relative importance of each of these models to explain the variation observed in the data was evaluated by AIC weights. Input data and scripts are available in the [Link], [Link].

### 2.7 Species richness per time

To estimate the species richness per time (*i.e*., the number of species at every millions of year) accounting for incomplete taxon sampling, we inferred the species richness confronting it with the mean crown age estimated in our time‐calibrated analysis for major subclades of Curimatidae. We included the 117 valid species of Curimatidae and categorized each of them as (1) geographic range only in lowland, (2) geographic range only in upland, or (3) geographic range in both lowlands and uplands. The categorization was based on the distribution of species visualized in the assembled geographic dataset (Figure 2a) and the literature for the unsampled taxa without information of geographic ranges (*e.g*., Vari, 1991, 1992, 2003). We estimated the number of species per million of year by dividing species counts by the mean crown age for seven major subclades of the Curimatidae: (1) *Curimatopsis*, (2) *Curimata*, (3) *Potamorhina*, (4) *Pseudocurimata*, (5) *Psectrogaster*, (6) *Cyphocharax*+*Curimatella*, and (7) *Steindachnerina*. For example, *Steindachnerina* has a total of 24 species (five only in lowlands, four only in uplands, and 15 widespread) and mean crown age of 29.1 Ma; the division of species per Ma indicates the presence of 0.171 lowland species/Ma, 0.137 upland species/Ma, 0.515 widespread species/Ma, and a total of 0.824 species/Ma. We merged *Cyphocharax* and *Curimatella* because the latter is polyphyletic with species nested in *Cyphocharax* (Melo et al., 2018). We also excluded *Cy. abramoides*, *Cy. nigripinnis*, and *Cy. multilineatus* from this analysis due to their phylogenetic placement outside the *Cyphocharax* + *Curimatella* clade (Melo et al., 2018).

## 3 RESULTS

### 3.1 Timing of curimatid diversification

**FIGURE 3:**
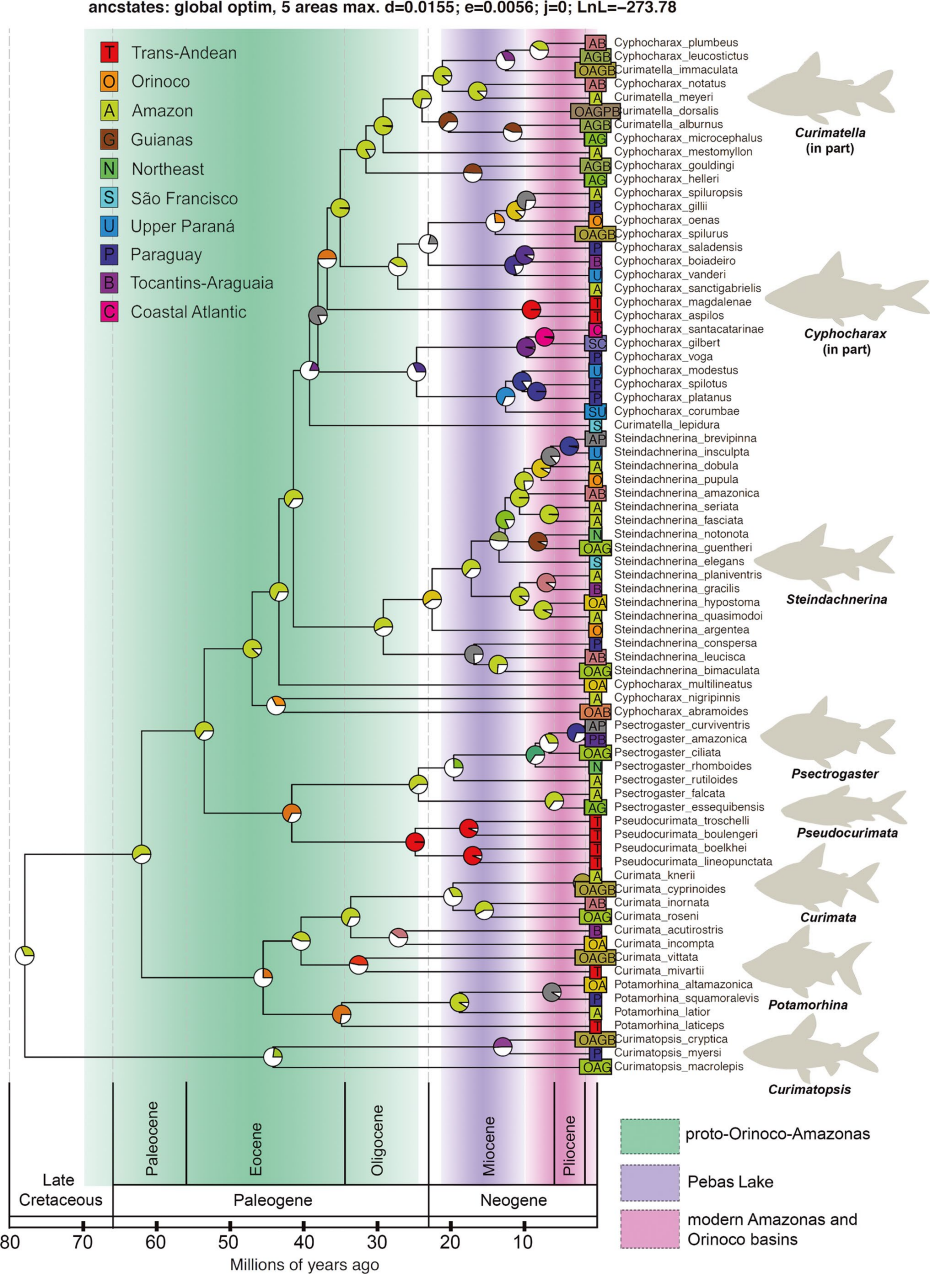
Time‐calibrated tree estimated in BEAST and ancestral range evolution of curimatids by bioregions estimated in BioGeoBEARS. The most likely ancestral state is colored in node circles, and less likely ancestral states are combined and shown in white color. Results show that the most recent common ancestor of all curimatids originated in the Late Cretaceous at around 78 Ma (90–66 Ma 95% HPD) in the lowlands of the Proto–Orinoco–Amazonas basin with multiple dispersal events to uplands of the Brazilian and Guiana shields

Our time‐calibrated analysis showed a Late Cretaceous split of Curimatidae and Chilodontidae *ca*. 79 Ma (91–68 Ma, 95% HPD) (Figure 3; [Link], [Link]) and *Curimatopsis* splitting from all remaining curimatids right after that, which represents the crown age of curimatids *ca*. 78 Ma (90–66 Ma, 95% HPD). Subsequent diversification occurred in the Paleogene, including the split of *Curimata* and *Potamorhina* (55–35 Ma, 95% HPD), and the split of *Psectrogaster* and *Pseudocurimata* (50–32 Ma, 95% Ma). The species‐rich *Cyphocharax sensu lato* clade diversified during the Eocene (54–39 Ma, 95% HPD), with the early divergence of *Cy. abramoides*, *Cy. nigripinnis*, and *Cy. multilineatus* (46–43 Ma, 95% HPD), the early diversification within *Steindachnerina* (36–22 Ma, 95% HPD), and the origin of the *Curimatella alburna* clade (37–25 Ma, 95% HPD) and of the *Cy. spilurus* clade (34–20 Ma, 95% HPD) occurring in the Eocene–Oligocene. Most internal cladogenesis occurred during the Miocene as, for example, the diversification within the *Curimata cyprinoides* clade (26–13 Ma, 95% HPD), within the *S. dobula* clade (17–10 Ma, 95% HPD), within the *Cy. gilbert* clade (26–23 Ma, 95% HPD), and inside the *Curimatella alburna* clade (<31 Ma) and the *Cy. spilurus* clade (<27 Ma) (Figure 3). The time‐calibrated tree including all node ages is available in the [Link], [Link].

### 3.2 Range evolution in river basins

The bipartite network clustering in the Infomap Bioregions delimited 10 bioregions (Figure 2c). Most bioregions closely resemble the areas of endemism hypothesized by Vari (1988) (Figure 2b), with two exceptions: (1) the recognition of a bioregion in the western Amazon Piedmont, which is not identified as a biogeographic region by Vari (1988), and (2) the union of Orinoco and Guianas as a single bioregion (Figure 2c). Even though our spatial delimitation suggests the union of Orinoco and Guianas, we assumed them as two bioregions in downstream biogeographic estimates to increase the possibilities of ancestral ranges. This reconstruction is also similar to a recent study incorporating geographic data of NFF from all over the Neotropics, which delimited additional smaller endemism areas, especially in the shields (Dagosta et al., 2020). Other minor discrepancies occurred due to grid‐cell limitations, such as the inclusion/exclusion of some trans‐Andean basins, and those of Northeastern Brazil and the Paraguay basin (Figure 2c).

Maximum‐likelihood estimates of geographic range evolution using DIVA model settings in BioGeoBEARS estimated that the MRCA of Curimatidae likely inhabited the Amazon basin (Figure 3). However, this estimate is supported by probability values below 50% likely due to the lack of additional species of *Curimatopsis*, the sister clade of all remaining genera, in which nine out of 11 species inhabit lowlands of the Amazon basin. Importantly, the MRCA of all other genera (except *Curimatopsis*) and subsequent intergeneric diversification had ancestral reconstructions estimated in the Amazon basin with probability values greater than 50% (Figure 3). Dispersal events from the Amazon to other bioregions began in the Paleocene, with three vicariant events between the Amazonian lowlands and trans‐Andean rivers that isolated lineages of either side of the Andes Cordillera, and one vicariant event between Orinoco and Trans‐Andean rivers (*Curimata mivartii* and *C. vittata*) during the Eocene–Oligocene (43–23 Ma, 95% HPD) (Figure 3). These four events occurred during the Eocene–Oligocene (55–24 Ma), therefore pre‐dating the complete separation of the Andean region by the Eastern Cordillera at around 12–9 Ma (Albert, Craig, et al., 2018; Hoorn et al., 2010). Species of curimatids achieved the Orinoco basin almost exclusively by biotic dispersal events from the Amazon lowlands (*e.g*., *Curimatopsis*, *Curimata*, *Steindachnerina argentea*, and *S. dobula*).

Phylogenetic and biogeographic data from curimatids show that most tropical South American basins are historically mixed areas, composed of divergent lineages of different origins, and recruited in separate ages (Dagosta & de Pinna, 2017). One example is the Araguaia–Tocantins system that comprises lineages from ancestral species inhabiting both lowland systems of the Amazon (*e.g*., *Curimata acutirostris* 37–17 Ma, 95% HPD; *Steindachnerina gracilis* 10–4 Ma, 95% HPD) and Paraguay (*Psectrogaster curviventris* 4.5–1.6 Ma, 95% HPD; *Cyphocharax boiadeiro* 15–5 Ma, 95% HPD) basins during the Miocene and Pliocene (24–1.8 Ma). The São Francisco basin has species acquired from Coastal Atlantic rivers of eastern Brazil (*Cy. gilbert* 11–4 Ma, 95% HPD) or the Upper Paraná (*Cy. corumbae* 20–7 Ma, 95% HPD), with an early dispersal during the Eocene (*Curimatella lepidura* 45–32 Ma, 95% HPD) (Figure 3).

The colonization of the Paraguay river occurred at least seven times in distinct time frames, however, always in clades originating from Amazon lowlands. The oldest event from Amazon lowlands to Paraguay basin occurred during the Late Oligocene (MRCA of the *Cyphocharax gilbert* clade 26–24 Ma, 95% HPD), with other dispersals dated to the Miocene (*Curimatopsis myersi* 20–7 Ma, 95% HPD; *Potamorhina squamoralevis* 10–3 Ma, 95% HPD; MRCA of the *Steindachnerina leucisca* clade 23–11 Ma, 95% HPD). Species from the Coastal Atlantic rivers of eastern Brazil derived from the Paraguay and Upper Paraná basins (*Cy. voga* (*Cy. gilbert*, *Cy. santacatarinae*)) during the Late Miocene (26–24 Ma, 95% HPD). The colonization of the Northeastern Brazil occurred from lowland Amazon basin during the Late Miocene (*Psectrogaster rhomboides* 12–5 Ma, 95% HPD; *S. notonota* 12–4 Ma, 95% HPD) (Figure 3). The MRCA of *Pseudocurimata* colonized the trans‐Andean region from the Orinoco basin (Figure 3).

### 3.3 Sympatric and vicariant events

The number of within‐basin and among‐basin cladogenetic events is similar within curimatids (Figure S1). This result may reflect the grouping of many lowland basins (*e.g*., Amazon basin) as a single bioregion rather than partitioned into multiple biogeographic units (see Dagosta & de Pinna, 2017). Within‐basin speciation events were more common within lowland than upland basins, and among‐basin speciation events are about equally distributed in lowland/lowland and lowland/upland river basins. Among within‐basin events, the most common was, by far, within the Amazon lowlands, followed by Trans‐Andean and Paraguay river basins (Figure S1). The most common among‐basin events were between Amazon/Paraguay followed by four transitions involving the Amazon basin: the vicariant events in Amazon/Araguaia–Tocantins, Amazon/Orinoco, Amazon/Trans‐Andean, and Amazon/Guianas (Figure S1).

### 3.4 From lowlands to uplands

**FIGURE 4:**
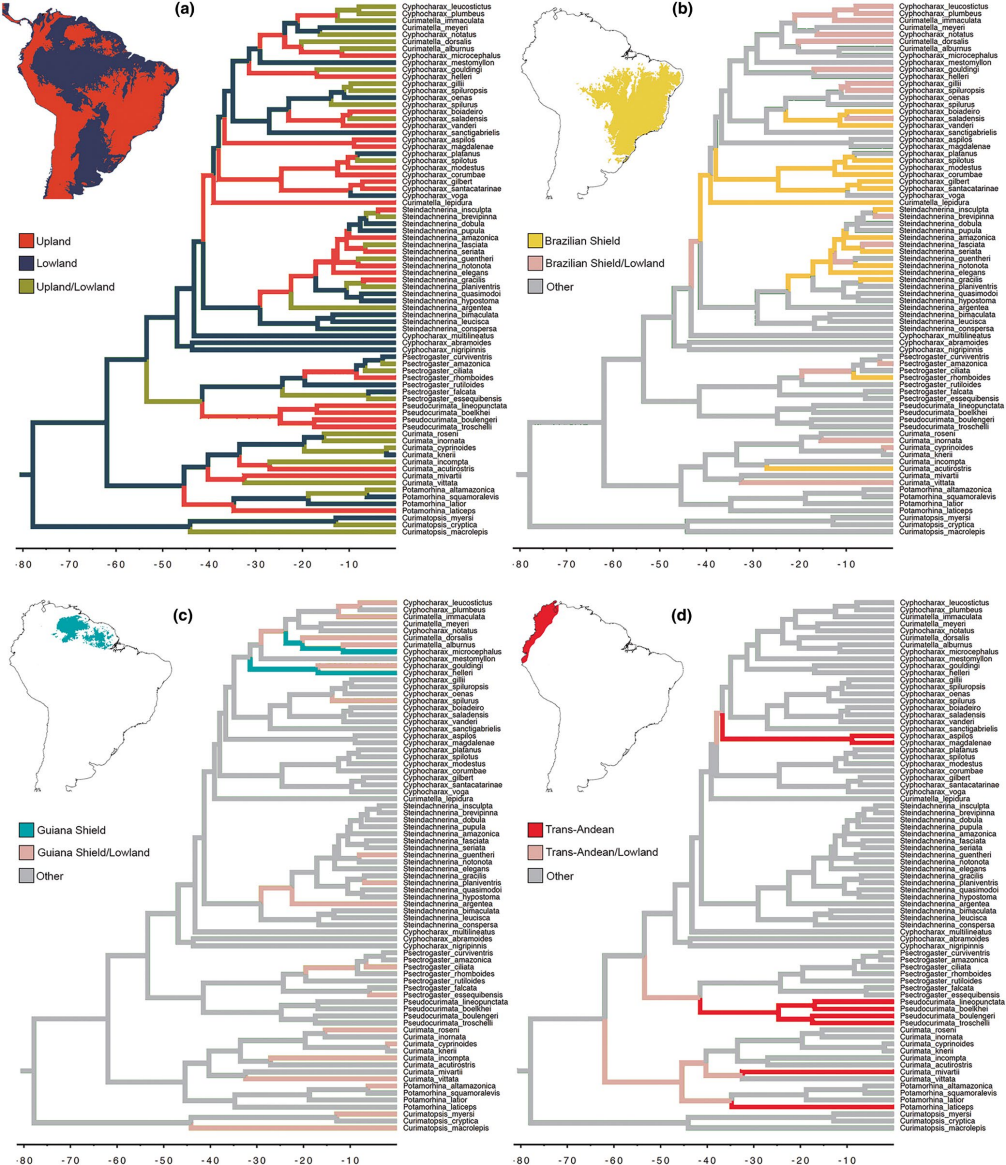
Ancestral‐area estimates of curimatid lineages during the Late Cretaceous and early Paleogene (*ca*. 80–50 Ma) according to BioGeoBEARS. (a) Species classified to river basins of lowlands (<250–300 m) and uplands (<250–300 m). (b) Species classified to river basins of the Brazilian Shield. (c) Species classified to river basins of the Guiana Shield. (d) Species classified to trans‐Andean region of northwestern South America. Faunas of upland basins are estimated to have independent events of dispersal during the middle Cenozoic (*ca*. 50–10 Ma)

Using the best‐fitting model (DIVA) setting in BioGeoBEARS, the MRCA of Curimatidae is estimated to have originated in lowland river basins during the Late Cretaceous to Paleogene (80–50 Ma, 95% HPD) and that multiple clades endemic to upland rivers have originated during the Early/Middle Eocene (55–36 Ma, 95% HPD) (Figure 4; [Link], [Link]). These phylogenetic and biogeographic patterns also show that upland taxa are polyphyletic relative to lowland taxa (Figure 4a), with multiple dispersal events from lowlands to the Brazilian Shield (Figure 4b), from lowlands to the Guiana Shield (Figure 4c), and from lowlands to the trans‐Andean region (Figure 4d).

The dispersal events from lowlands to the Brazilian Shield occurred during the Paleogene and Neogene (Figure 4b). Two Paleogene dispersals occurred during the Eocene–Oligocene, one of *Curimatella lepidura* to São Francisco (45–32 Ma, 95% HPD) and another of the MRCA of the *Cyphocharax gilbert* clade to Paraguay and Upper Paraná (44–32 Ma, 95% HPD). Dispersal events near the Paleogene–Neogene boundary occurred in *Curimata acutirostris* to Araguaia–Tocantins (37–17 Ma, 95% HPD) and in the clade (*Cy. vanderi* (*Cy. saladensis*, *Cy. boiadeiro*)) to Paraguay, Upper Paraná, and then to the Upper Araguaia (29–17 Ma, 95% HPD). Subsequently, two Neogene dispersal events promoted the colonization from lowlands to Northeastern Brazil (*Psectrogaster rhomboides* 12–5 Ma, 95% HPD), and from lowlands to the Upper Paraná (*Steindachnerina insculpta* 5–2.5 Ma, 95% HPD). At least twelve more dispersal events occurred to the Brazilian Shield with species persisting in the original lowlands, especially in *Cyphocharax* and *Curimatella* (Figure 4b).

Two dispersals during the Eocene–Oligocene boundary occurred from lowlands to the Guiana Shield: one of the MRCA of *Cyphocharax helleri* and *Cy. gouldingi* (37–25 Ma, 95% HPD), and another of the MRCA of the clade (*Curimatella dorsalis* (*Cu. alburna*, *Cy. microcephalus*)) (29–18 Ma, 95% HPD). At least 14 more dispersals occurred to the Guiana Shield with species maintaining the original distribution in lowlands (Figure 4c). Finally, during the Paleogene (Eocene and Oligocene), four dispersal events occurred to the area today named as trans‐Andean region. Those are the lineages of *Potamorhina laticeps* in the Lago Maracaibo (45–25 Ma, 95% HPD), *Curimata mivartii* in the Río Magdalena (43–23 Ma, 95% HPD), the MRCA of all six species of *Pseudocurimata* in the coastal rivers from northwestern Peru to Río Atrato in Colombia (50–32 Ma, 95% HPD), and the *Cy. magdalenae* clade in the Maracaibo and Magdalena–Cauca (43–30 Ma 95% HPD), being the only curimatid reaching the lower Central America (Figure 4d). The fifth trans‐Andean clade, *Steindachnerina atratoensis* from Río Atrato, was unfortunately unavailable in the present study. All those four clades have species endemic to the trans‐Andean river systems (Figure 4d).

### 3.5 Diversification rates

**FIGURE 5:**
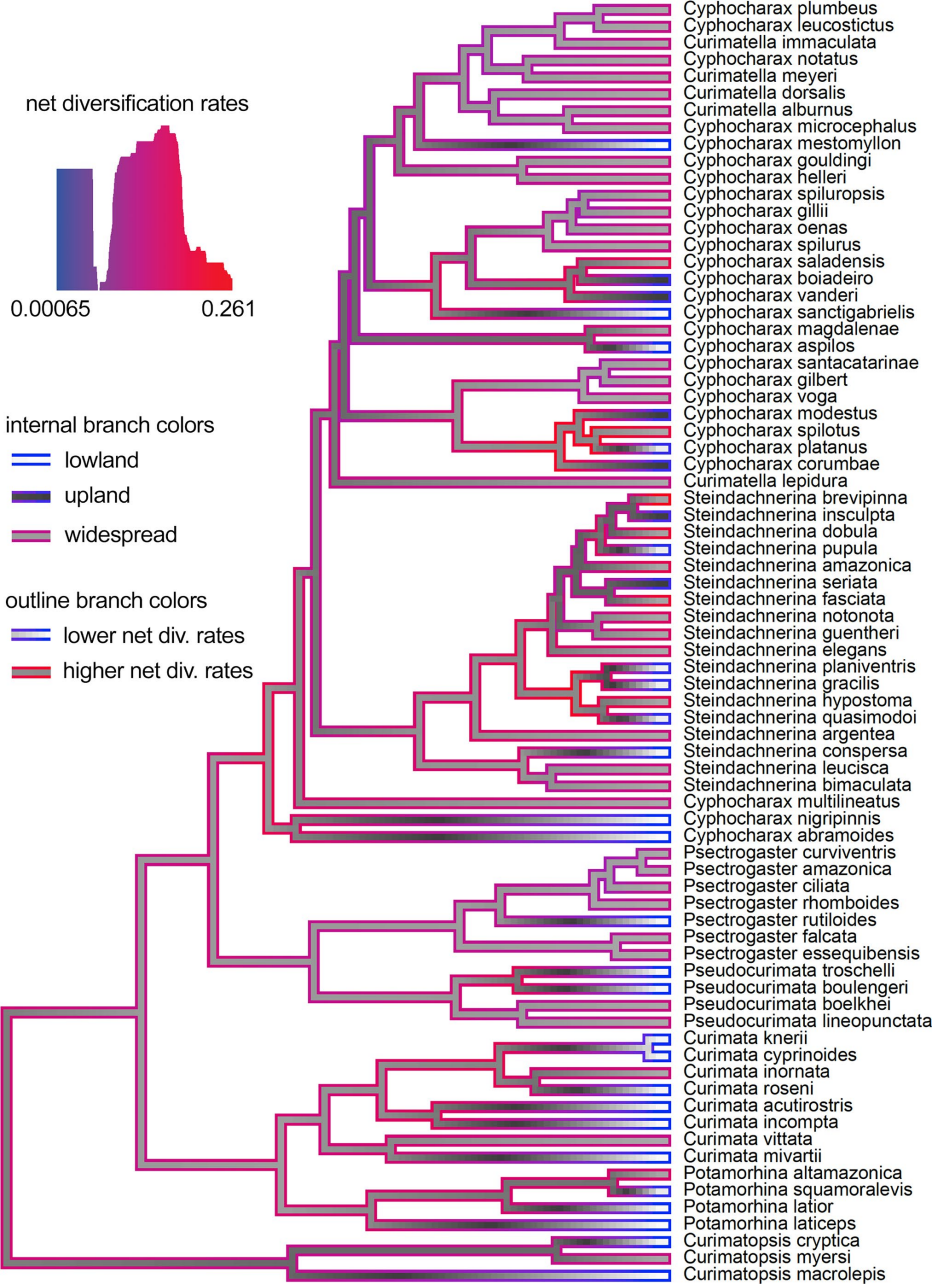
GeoHiSSE range‐dependent diversification plot of net diversification rates (M4, range‐dependent process with hidden states). Colors of the branch outlines (blue to red) indicate the distribution of net diversification rates along the topology (<0.001–0.26). Higher net diversification rates are prevalent in widespread taxa, especially in subclades of *Cyphocharax* and *Steindachnerina* (bright red outlines)

The range‐dependent diversification analysis (GeoSSE/GeoHiSSE) tested four distinct models: M1 (null model) assumes a homogeneous diversification rate across the tree independent of the ranges; M2 (GeoSSE) assumes a range‐dependent diversification process without hidden states; M3 (GeoHiSSE) assumes shifts in diversification across the tree independent of the ranges; and M4 (GeoHiSSE) assumes a range‐dependent diversification process with hidden and multiple rate states (Caetano et al., 2018). We computed the relative importance of each of the models to explain the variation observed in the data using AIC weights. Results indicate that the best‐fitting model was M4 (Table 2), where variation in net diversification rates is correlated with geographic ranges (in our case, lowlands and uplands). The two best‐fitting models (M2 and M4) account together for 0.999 of the AIC weight (0.243 and 0.756, respectively), assuming shifts in diversification rates, and vary in whether hidden states are allowed or not. The two other models treating diversification and range evolution as independent processes appeared with poorer fits and presented AIC weights below 0.0001 (Table 2). We then performed a marginal reconstruction for each of the models in the set, reconstructed the hidden states at the nodes of the phylogeny, and plotted the results in the topology (Figure 5). Results indicate a range of 0.0006 to 0.261 for net diversification rates with higher rates occurring mostly in clades with widespread taxa (*i.e*., taxa occurring in both lowland and upland basins), as denoted by the yellow branches with bright red outlines (Figure 5). These are more evident in subclades of *Cyphocharax* and *Steindachnerina* with a few instances within *Curimata* and *Pseudocurimata* (Figure 5).

**TABLE 2:** Diversification rate models tested by GeoSSE/GeoHiSSE

	Model description	Range effect	Hidden states	AIC weights	AIC (%)
M1	GeoSSE, no range effect, no hidden state effect, null model	No	No	2.071081e−05	0.002
M2	GeoSSE, range effect, no hidden state effect (original GeoSSE model)	Yes	No	2.432576e−01	24.3
M3	GeoHiSSE, no range effect, hidden state effect	No	Yes	2.802921e−06	<0.001
M4	GeoHiSSE, both range and hidden state effect	Yes	Yes	7.567189e−01	75.6

Abbreviation: AIC, Akaike information criterion.

### 3.6 Species richness per time

In addition, we accounted for unsampled taxa by calculating the total number of species per million of years for seven major subclades taking our time‐calibrated tree as a chronological framework. In lowland regions, rates of species per time are higher for the *Cyphocharax* + *Curimatella* clade (0.306 species per Ma) and for *Curimata* (0.198 species per Ma). In upland regions exclusively, the species accumulation is higher for *Cyphocharax* + *Curimatella* (0.230 species per Ma) and *Steindachnerina* (0.137 species per Ma). For widespread species occurring in both settings (lowlands and uplands), there is significantly higher accumulation of species in *Cyphocharax* + *Curimatella* (0.664 species per Ma) and *Steindachnerina* (0.515 species per Ma). The total number of species per time without geographic categorization indicates elevated numbers in the clade *Cyphocharax* + Curimatella (1.202 species per Ma), followed by *Steindachnerina* (0.824 species per Ma), and with a total average of 0.473 curimatid species per Ma in the Neotropics (*i.e*., approximately one species every two million years) (Table 3).

**TABLE 3:** Rates of total species richness per time of diversification in seven subclades of Curimatidae

Subclade	Total species richness	Mean crown age (Ma)	Lowland species per Ma	Upland species per Ma	Widespread species per Ma	Total species per Ma
*Curimatopsis*	11	44.1	0.158	0.045	0.045	0.249
*Curimata*	13	40.3	0.198	0.000	0.124	0.322
*Potamorhina*	5	34.7	0.115	0.000	0.028	0.144
*Pseudocurimata*	6	24.7	0.080	0.000	0.161	0.242
*Psectrogaster*	8	24.3	0.041	0.041	0.246	0.329
*Cyphocharax* + *Curimatella*	47	39.1	0.306	0.230	0.664	1.202
*Steindachnerina*	24	29.1	0.171	0.137	0.515	0.824
Average	–	–	0.152	0.064	0.254	0.473

Note

Mean crown age based on our time‐calibrated phylogeny. Total species richness includes the number of all valid species for each clade. The *Cyphocharax* + *Curimatella* clade does not include the early‐branching species *Cy. abramoides*, *Cy. nigripinnis*, and *Cy. multilineatus*.

Abbreviation: Ma, millions of years ago.

## 4 DISCUSSION

### 4.1 Cretaceous origins and Cenozoic diversification

The time calibration and ancestral biogeographic estimations indicate an origin of curimatids in lowlands of the Proto–Orinoco–Amazon (POA) basin during the Late Cretaceous (*ca*. 78 ± 12 Ma) (Figures 3, 4). This divergence time estimate is older than previously calibrated phylogenies for characiform fishes, which estimated Paleogene stem (*ca*. 55–52 Ma) and crown (*ca*. 50–37 Ma) ages for all curimatids (Burns & Sidlauskas, 2019; Melo et al., 2021). A Late Cretaceous origin for Curimatidae is consistent with the known fossil record. The edentulous dentary fragment of †*Eotexachara* tentatively assigned to Curimatidae was recently described from the early Campanian strata (Late Cretaceous ~81 Ma) of the Aguja Formation in West Texas (Wick, 2021). These new fossil and molecular‐dating evidence set the approximate origin of Curimatidae for the Late Cretaceous. Furthermore, the curimatid †*Cyphocharax mosesi* from the Oligocene–Miocene boundary of the Tremembé Formation (*ca*. 28–20 Ma) has been tentatively discussed to be closer to extant species of the *Cyphocharax gilbert* clade than to other modern *Cyphocharax* or *Steindachnerina* (Malabarba, 1996; Melo et al., 2018). The presence of †*Cyphocharax mosesi*, a fossil phylogenetically close to modern species near the Oligocene–Miocene boundary, suggests that at least some modern curimatid genera were already present by that time period. This perspective is also supported by the presence of †*Plesiocurimata* in the same Tremembé Formation, which may be phylogenetically closer to a clade of most derived taxa (*Curimatella*, *Cyphocharax*, *Steindachnerina*) and not the plesiomorphic sister group to all other curimatids as previously suggested (Figueiredo & Costa‐Carvalho, 1999).

Most species‐level diversification within curimatid genera is estimated to have occurred during the Late Cenozoic (Figures 3, 4, 5), in association with the formation of the river courses and major aquatic habitat types of the modern Amazon basin. For example, the crown MRCA of *Steindachnerina* is dated to the Oligocene (*ca*. 29 Ma), after which this clade accumulated its 24 known valid species. The clade including *Cyphocharax* and *Curimatella* (Figure 3) is represented by 47 valid species that diversified since the Late Eocene (*ca*. 38 Ma), at a rate of approximately 1.2 species per Ma (Table 3), although the actual diversity of this and other clades seems to be underestimated (Melo et al., 2016). We hypothesize that diversification of these clades may have been triggered by: (1) large river captures promoting dispersal from POA to adjacent upland drainages of the Guiana and Brazilian shields (Albert, Val, et al., 2018; Tagliacollo et al., 2015); (2) derived morphophysiological or behavioral adaptations that allowed members of colonizing populations access to novel food or habitat resources in upland basins (Silva et al., 2016), and/or (3) reduced competition and predation in lower diversity rivers of the upland basins (Burns & Sidlauskas, 2019; López‐Fernández et al., 2013). Furthermore, some of the large drainage basins adjacent to the modern Western Amazon, for example, the Upper Madeira and Paraguay basins, share many similarities in overall climate, water chemistry, and habitat physiognomy that may have facilitated geographic range expansions and population establishment (Carvalho & Albert, 2011).

### 4.2 Species accumulation in lowlands

Initial diversification of curimatid lineages and phenotypes occurred in lowland Neotropical river basins during the Late Cretaceous, with diversification into clades with modern phenotypes and geographic ranges during the Paleogene and Neogene. This partitioning of the continent into core lowlands and peripheral uplands served to amplify the initial lineage diversity, generating the extraordinary levels observed in modern faunas. Low extinction rates have also been posited as a driver of high net diversification in NFF taxa (Lundberg et al., 1998), with most known paleofaunas having achieved modern phenotypes by the Late Miocene (Albert, Val, et al., 2018; Hoorn et al., 2010). However, extinction rate estimates are poorly constrained in the absence of a robust paleontological record (Beaulieu & O'Meara, 2015; Rabosky, 2010, 2016), and tropical rivers are poor environments for fossil formation and discovery (López‐Fernández & Albert, 2011). Our hypothesis that Neotropical lowlands served as macroevolutionary museums is mainly supported by neontological evidence from the time‐calibrated molecular phylogeny, showing accumulation of curimatid species in the lowlands (Figures 3, 4).

Previous studies using macroevolutionary models have shown similar results. A study with the New World avifauna shows that tropical regions act as museums of diversity accumulating species diversity in low extinction rates (Gaston & Blackburn, 1996). In a molecular phylogenetic study of leaf beetles (Coleoptera: Chrysomelidae), McKenna and Farrell (2006) presented evidence that Neotropical forests are both museums and cradles of diversity showing distinct events of species diversification during the Cenozoic. The same pattern appears in other clades, as, for example, ants (Hymenoptera: Formicidae) that diversified during the Cretaceous were affected by the Cretaceous–Paleogene extinction, and experienced high diversification rates more during the Paleogene and Neogene (Moreau & Bell, 2013). More recently, a macroevolutionary study indicated bursts of speciation in three characiform families (Anostomidae, Serrasalmidae, and Characidae) around the Oligocene (~30 Ma), suggesting a cradle model of higher diversification rates driving the appearance of a remarkable species diversity in characoid fishes (Melo et al., 2021). The results found herein agree with those studies and emphasize the key role of the Amazon lowlands as a primary source of Neotropical biodiversity for many biotas (Antonelli, Ariza, et al., 2018; Antonelli, Zizka, et al., 2018).

### 4.3 Multiple dispersals to upland basins

Contrasting with lowlands, the cratonic uplands of the Guiana and Brazilian shields are better described as sinks of diversity, receiving multiple dispersal events of curimatid fishes during the Cenozoic, especially those of the most species‐rich genera *Cyphocharax* and *Steindachnerina*, but also relevant dispersals of *Curimata*, *Curimatella*, and *Pseudocurimata* with closely related species in trans‐Andean regions (Figure 4). These multiple dispersals from lowland to upland basins occurred mostly during the Eocene and Oligocene, with a few events during the Neogene (Figures 3, 4) associated with higher net diversification rates of widespread taxa (Figure 5). These results reject previous hypotheses of origin and expansion of faunas from cratonic upland to lowland basins (Albert & Carvalho, 2011; Eigenmann & Allen, 1942). Instead, our results support a pattern of biotic turnover potentialized by mega river captures in lowland basins that catalyzed subsequent events of allopatric speciation in large river basins draining the upland shields (Albert, Craig, et al., 2018; Albert et al., 2011; Lundberg et al., 1998).

The lowland origin in the POA followed by multiple independent colonizations of upland rivers is also supported by recent biogeographic reconstructions in other NFF clades. Long‐whiskered catfishes (Pimelodidae), for example, emerged during the Late Cretaceous in the sub‐Andean foreland basin, and had diversification directly affected by three major river captures between the Amazon and La Plata lowlands (Tagliacollo et al., 2015). Armored suckermouth catfishes (Hypostominae) originated during the Eocene in the POA, dispersed to uplands of the Paraná river basin, and experienced accelerated rates of speciation and ecomorphological diversification during the Miocene (Cardoso et al., 2012; Silva et al., 2016). Species of the characiform clades *Salminus* (Bryconidae) and *Schizodon* (Anostomidae) and freshwater stingrays (Potamotrygoninae) represent other examples of dispersal from the Proto–Amazon to upland basins of the Brazilian and Guiana Shield, such as Araguaia–Tocantins, La Plata, São Francisco, Parnaíba, and Xingu, among others (Fontenelle et al., 2021; Machado et al., 2018; Ramirez et al., 2020). In curimatids, lowland river captures between the Amazon/Paraguay and the Amazon/Tocantins (Figure 3, Figure S1) facilitated the dispersal of taxa especially into the Brazilian Shield, although evidence for biotic or abiotic factors driving the upland colonization is not yet available, nor why those ancient lineages dispersed to uplands only in the Cenozoic.

### 4.4 Comparisons with previous reconstructions and conclusions

Among several conclusions advanced by Vari (1988), we corroborate (1) the Amazon–Orinoco–Guianas and the Amazon–Paraguay associations, (2) four vicariant events during the Eocene across the Andes (five in Vari's hypothesis), (3) numerous cladogenetic events prior to uplift of the northern Andes, and (4) colonization of the São Francisco and northeastern Brazil curimatid faunas from different adjacent bioregions. In addition, the results show, consistent with the vicariance model (Vari, 1988), that curimatids diversified in lowlands of the POA and Proto–Paraguay river basins, especially during the Early and Middle Miocene in the Pebas mega‐wetland system of the modern Western Amazon (Albert, Val, et al., 2018; Hoorn et al., 2010). The role of lowland river captures between the POA and Proto–Paraguay leading to vicariance and geodispersal reinforces recent biogeographic reconstructions and interspecific relationships of NFF taxa from the Madeira and Paraguay basins (Carvalho & Albert, 2011; Dorini et al., 2020; Hubert et al., 2007; Mateussi et al., 2017, 2020; Melo et al., 2016; Ramirez et al., 2020; Tagliacollo et al., 2015), and the recent biogeographic reconstruction of more than 4760 Neotropical fishes despite small‐scale differences (Dagosta et al., 2020). The discrepancy relative to this specific study is that curimatids are more informative on a larger biogeographic scale, likely due to the fewer narrowly distributed species compared with other species‐rich groups such as characids (Characiformes), loricariids (Siluriformes), and rivulids (Cyprinodontiformes) (Dagosta et al., 2020).

Although the phylogenetic hypothesis of the present study is based on genetic information from six genes and about two‐thirds of all known curimatid species, it is possible to test these results with studies using more complete taxon and gene sampling. For example, the inclusion of more lowland species of *Curimatopsis* might strengthen the signal of the lowland origin for the MRCA of all curimatids in the ancestral state reconstruction, the addition of *Steindachnerina atratoensis* will likely recognize the fifth event of dispersal from POA to the trans‐Andean region, and the improvement of the phylogenetic resolution at the base of *Cyphocharax sensu lato* may refine the evolutionary and biogeographic history of that clade. However, the addition of taxon/gene sampling is not expected to change the general results regarding lowland origins with multiple dispersals to uplands. Finally, our results demonstrate that molecular phylogenetic and parametric biogeographic studies can recognize similar biogeographical patterns when compared to more descriptive biogeographic studies (Vari, 1988). The coherence also reflects the predictive power of historical biogeography, which, regardless of the method, still exposes the intricate correlations among geomorphological processes and evolutionary relationships among Neotropical freshwater fishes.

## CONFLICT OF INTEREST

The authors declare that they have no known competing financial interests or personal relationships that could have appeared to influence the work reported in this paper.

## AUTHOR CONTRIBUTIONS

**Bruno F. Melo:** Conceptualization (equal); Data curation (equal); Formal analysis (equal); Funding acquisition (equal); Investigation (equal); Methodology (equal); Project administration (equal); Validation (equal); Visualization (equal); Writing‐original draft (equal); Writing‐review & editing (equal). **James S. Albert:** Conceptualization (equal); Investigation (equal); Methodology (equal); Validation (equal); Visualization (equal); Writing‐review & editing (equal). **Fernando D'Agosta:** Data curation (equal); Formal analysis (equal); Investigation (equal); Methodology (equal); Visualization (equal); Writing‐review & editing (equal). **Victor A. Tagliacollo:** Conceptualization (equal); Data curation (equal); Formal analysis (equal); Methodology (equal); Visualization (equal); Writing‐review & editing (equal).

### OPEN RESEARCH BADGES

This article has been awarded <Open Materials, Open Data> Badges. All materials and data are publicly accessible via the Open Science Framework at [https://doi.org/10.5061/dryad.9p8cz8wfw].

## Supporting information

Fig S1

Table S1

## Data Availability

[Link], [Link] is available online. Matrices, scripts, tables, trees, and other files are deposited in the Dryad Digital Repository: https://doi.org/10.5061/dryad.9p8cz8wfw.
