# Supplementary figures and images for: Biogeography of curimatid fishes reveals multiple lowland–upland river transitions and differential diversification in the Neotropics (Teleostei, Curimatidae)
Source: Ecol Evol. 2021 Nov 9;11(22):15815–32. doi: 10.1002/ece3.8251 (PMC8601890; doi:10.1002/ece3.8251)

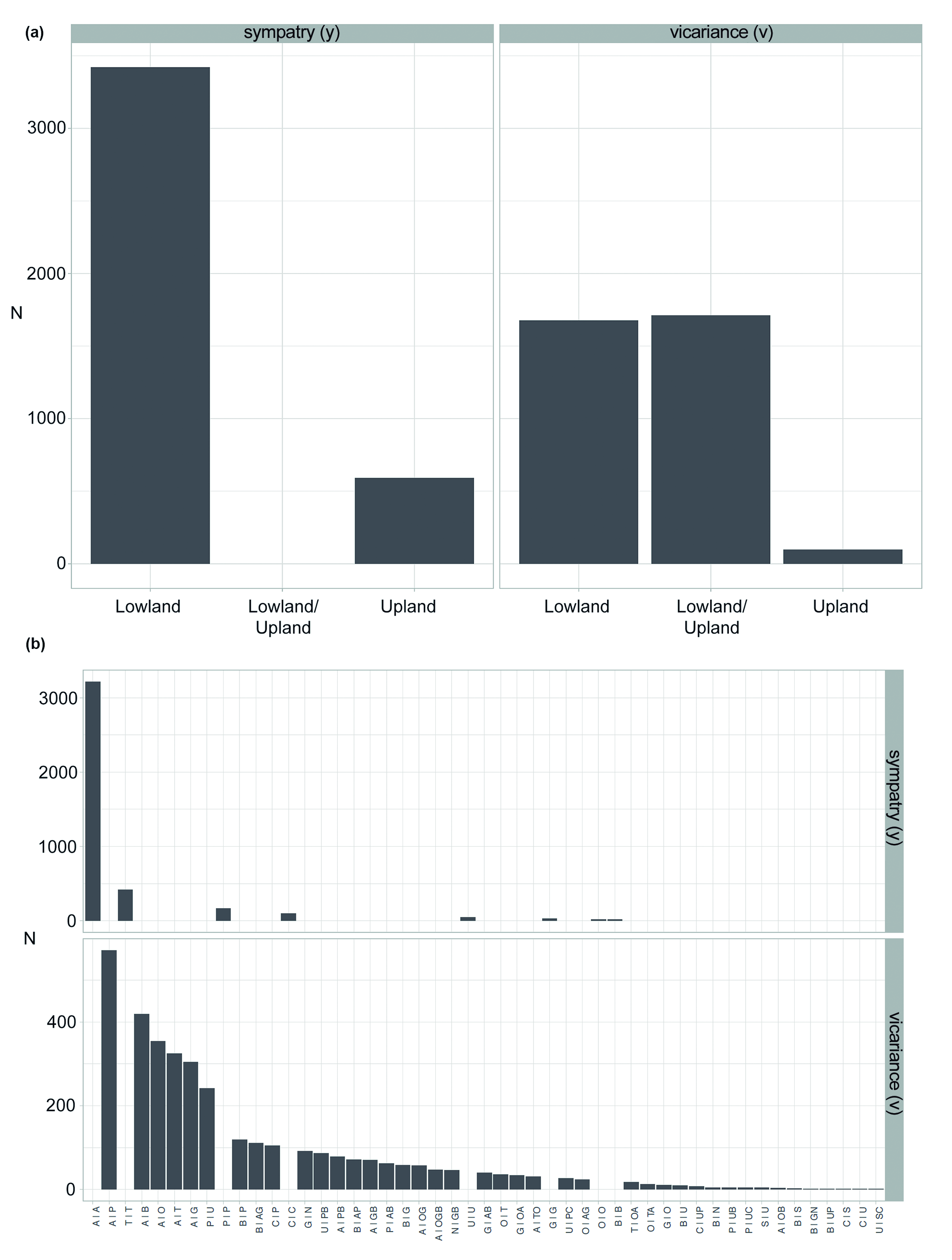

Supplement: Supplementary file 1 — Fig S1 [file ECE3-11-15815-s002.tif]
