# Supplementary material for: Biogeography of curimatid fishes reveals multiple lowland–upland river transitions and differential diversification in the Neotropics (Teleostei, Curimatidae)
Source: Ecol Evol. 2021 Nov 9;11(22):15815–32. doi: 10.1002/ece3.8251 (PMC8601890; doi:10.1002/ece3.8251)
Supplement: Supplementary file 2 — Table S1 [file ECE3-11-15815-s001.docx]

**Table S1.** Taxon and voucher information for the analyzed species with GenBank accession numbers for each locus. Institution acronyms follow Sabaj (2016). Additional locality information available in Melo et al. (2018).

| Species | Voucher | Tissue | *16S* | *COI* | *Cytb* | *Myh6* | *Rag1* | *Rag2* |
| --- | --- | --- | --- | --- | --- | --- | --- | --- |
| *Curimata acutirostris* | LBP 1802 | 13168 | MH537105 | MH537229 | - | MH537487 | MH542683 | MH542797 |
| *Curimata acutirostris* | LBP 7744 | 36724 | MH537106 | MH537230 | MH537400 | MH537522 | MH542711 | - |
| *Curimata acutirostris* | LBP 9260 | 41319 | MH537107 | MH537231 | MH537407 | MH537529 | MH542722 | MH542817 |
| *Curimata* aff. *cyprinoides* | LBP 5430 | 27169 | - | - | MH537390 | MH537513 | MH542717 | - |
| *Curimata cyprinoides* | MHNG 2705.006 | SU07-217 157-17 | MH537108 | MH537232 | MH537363 | MH537489 | - | MH542799 |
| *Curimata cyprinoides* | USNM 402473 | GY11-1-03 | KX087054 | KX086781 | KX086803 | KX086907 | KX086961 | KX086978 |
| *Curimata incompta* | ANSP 191354 | BO-6125 | MH537109 | MH537234 | MH537449 | MH537569 | MH542762 | MH542847 |
| *Curimata inornata* | LBP 13842 | 57293 | MH537110 | MH537235 | MH537428 | MH537548 | MH542736 | MH542832 |
| *Curimata inornata* | USNM 402471 | GY11-2-53 | MH537111 | MH537236 | MH537451 | MH537570 | MH542764 | MH542848 |
| *Curimata knerii* | LBP 12864 | 53467 | MH537112 | MH537237 | MH537422 | MH537543 | MH542732 | MH542827 |
| *Curimata knerii* | LBP 12864 | 53468 | MH537113 | MH537238 | MH537423 | MH537544 | MH542746 | MH542828 |
| *Curimata mivartii* | CMIV-UNAL-001 | UNAL001 | KP025764 | KP025764 | KP025764 | - | - | - |
| *Curimata roseni* | ANSP 189094 | V071 | MH537114 | MH537239 | MH537483 | MH537591 | - | MH542864 |
| *Curimata roseni* | ANSP 180389 | T38 | MH537115 | MH537240 | MH537481 | MH537590 | MH542793 | MH542863 |
| *Curimata vittata* | LBP 13846 | 57302 | - | MH537241 | MH537429 | MH537549 | MH542737 | - |
| *Curimata vittata* | ANSP 182217 | V070 | - | MH537242 | MH537482 | - | MH542794 | - |
| *Curimatella alburna* | ANSP 182223 | T2177 | MH537116 | - | MH537480 | - | MH542792 | - |
| *Curimatella dorsalis* | LBP 3054 | 19167 | MH537117 | MH537243 | MH537376 | MH537500 | - | MH542805 |
| *Curimatella dorsalis* | LBP 3759 | 22034 | HQ171290 | KF562433 | HQ289579 | HQ289000 | - | HQ289387 |
| *Curimatella dorsalis* | OS 18376 | PE10-069 | MH537118 | MH537244 | MH537457 | MH537575 | MH542771 | MH542851 |
| *Curimatella immaculata* | ANSP 191246 | BO6119 | MH537119 | MH537245 | MH537448 | MH537568 | MH542761 | MH542846 |
| *Curimatella immaculata* | LBP 5606 | 27341 | MH537120 | MH537246 | MH537392 | MH537515 | - | - |
| *Curimatella immaculata* | LBP 15269 | 63217 | MH537121 | MH537247 | MH537437 | MH537556 | MH542756 | MH542838 |
| *Curimatella lepidura* | LBP 11352 | 45506 | MH537122 | MH537248 | MH537414 | MH537535 | MH542729 | MH542824 |
| *Curimatella lepidura* | LBP 11352 | 45507 | MH537123 | MH537249 | MH537415 | MH537536 | MH542730 | MH542825 |
| *Curimatella meyeri* | LBP 4253 | 22748 | MH537124 | MH537250 | MH537385 | MH537508 | MH542703 | MH542807 |
| *Curimatella meyeri* | UFRO uncat | 67037 | MH537125 | MH537251 | MH537439 | MH537558 | MH542757 | MH542840 |
| *Curimatopsis cryptica* | LBP 19769 | 24361 | MH537126 | - | MH537387 | - | MH542715 | - |
| *Curimatopsis cryptica* | ANSP 189091 | 7057 | MH537127 | MH537252 | MH537446 | MH537566 | MH542759 | MH542845 |
| *Curimatopsis macrolepis* | ANSP 178188 | 1697 | KX087053 | KX086740 | KX086800 | KX086864 | KX086940 | KX086977 |
| *Curimatopsis macrolepis* | OS 18337 | PE10-086 | MH537128 | KU519386 | MH537459 | - | MH542772 | MH542853 |
| *Curimatopsis myersi* | LBP 14006 | 58310 | MH537130 | KU519360 | MH537430 | MH537550 | MH542738 | MH542833 |
| *Curimatopsis myersi* | LBP 14006 | 58311 | MH537131 | KU519361 | MH537431 | MH537551 | MH542739 | MH542834 |
| *Cyphocharax abramoides* | ANSP 189092 | V5411 | MH537132 | MH537253 | MH537484 | MH537592 | MH542795 | MH542865 |
| *Cyphocharax aspilos* | LBP 6109 | 29560 | HQ171363 | - | HQ289650 | HQ289071 | HQ289264 | - |
| *Cyphocharax aspilos* | LBP 6109 | 29561 | MH537133 | MH537254 | MH537394 | MH537517 | MH542707 | - |
| *Cyphocharax boiadeiro* | LBP 1446 | 12595 | - | MH537255 | MH537360 | MH537485 | MH542680 | - |
| *Cyphocharax boiadeiro* | LBP 1446 | 12596 | MH537134 | MH537256 | MH537361 | MH537486 | MH542682 | - |
| *Cyphocharax corumbae* | LBP 17244 | 68908 | MH537135 | MH537257 | MH537444 | MH537563 | MH542755 | - |
| *Cyphocharax corumbae* | LBP 17244 | 68909 | MH537136 | MH537258 | MH537445 | MH537564 | - | - |
| *Cyphocharax gilbert* | LBP 8343 | 40130 | KX087056 | KX086768 | KX086805 | KX086894 | KX086946 | - |
| *Cyphocharax gilbert* | LBP 10734 | 49733 | MH537137 | MH537259 | MH537420 | MH537541 | - | - |
| *Cyphocharax gillii* | LBP 10789 | 49895 | MH537138 | MH537260 | MH537421 | MH537542 | MH542731 | - |
| *Cyphocharax gouldingi* | LBP 2432 | 16291 | MH537139 | MH537264 | MH537367 | - | MH542690 | - |
| *Cyphocharax gouldingi* | MHNG 2664.100 | GF03-135 157-16 | MH537140 | MH537265 | MH537362 | MH537488 | MH542684 | MH542798 |
| *Cyphocharax helleri* | MHNG 2718.046 | SU08-489 157-24 | MH537141 | MH537266 | MH537365 | MH537491 | MH542687 | - |
| *Cyphocharax leucostictus* | LBP 15951 | 66102 | MH537142 | MH537267 | MH537438 | MH537557 | MH542753 | MH542839 |
| *Cyphocharax magdalenae* | STRI 1144 | BFD 1642 | MH537143 | MH537268 | MH537471 | MH537581 | MH542783 | - |
| *Cyphocharax magdalenae* | STRI 1145 | BFD 1631 | MH537144 | MH537269 | MH537472 | MH537582 | MH542784 | - |
| *Cyphocharax mestomylon* | LBP 16372 | 67271 | MH537145 | MH537270 | MH537440 | MH537559 | - | MH542841 |
| *Cyphocharax mestomylon* | LBP 16372 | 67272 | MH537146 | MH537271 | MH537441 | MH537560 | - | MH542842 |
| *Cyphocharax microcephalus* | MHNG 2723.019 | SUJM-129 157-25 | MH537147 | MH537272 | MH537366 | - | MH542689 | MH542800 |
| *Cyphocharax modestus* | LBP 8362 | 40165 | MH537148 | MH537273 | MH537404 | MH537526 | MH542723 | - |
| *Cyphocharax modestus* | LBP 9654 | 45695 | MH537149 | - | MH537416 | MH537537 | - | - |
| *Cyphocharax multilineatus* | LBP 6964 | 34001 | MH537150 | MH537274 | MH537396 | MH537518 | MH542709 | MH542812 |
| *Cyphocharax multilineatus* | LBP 6964 | 34002 | MH537151 | MH537275 | MH537397 | MH537519 | MH542719 | MH542813 |
| *Cyphocharax nigripinnis* | LBP 4490 | 24472 | MH537152 | MH537276 | - | MH537510 | MH542716 | MH542808 |
| *Cyphocharax notatus* | LBP 5734 | 26865 | MH537153 | MH537277 | MH537388 | MH537511 | MH542704 | MH542809 |
| *Cyphocharax notatus* | LBP 5734 | 26867 | MH537154 | MH537278 | MH537389 | MH537512 | MH542705 | MH542810 |
| *Cyphocharax oenas* | LBP 9970 | 46801 | MH537155 | MH537279 | MH537417 | MH537538 | MH542743 | - |
| *Cyphocharax oenas* | LBP 9970 | 46802 | MH537156 | MH537280 | MH537418 | MH537539 | MH542744 | - |
| *Cyphocharax platanus* | MCP 21655 | T21655 | MH537157 | MH537281 | MH537454 | MH537572 | MH542767 | - |
| *Cyphocharax plumbeus* | OS 18326 | PE10-145 | MH537158 | MH537282 | MH537467 | - | MH542778 | - |
| *Cyphocharax plumbeus* | OS 18326 | PE10-149 | MH537159 | MH537283 | MH537468 | MH537578 | MH542780 | MH542856 |
| *Cyphocharax plumbeus* | OS 18373 | PE10-071 | MH537160 | MH537284 | MH537458 | MH537576 | MH542770 | MH542852 |
| *Cyphocharax plumbeus* | OS 18769 | PE10-099 | MH537161 | MH537285 | MH537460 | - | MH542773 | - |
| *Cyphocharax saladensis* | LBP 13164 | 55055 | MH537162 | MH537286 | MH537425 | MH537546 | MH542734 | MH542830 |
| *Cyphocharax saladensis* | LBP 13164 | 55062 | MH537163 | MH537287 | MH537426 | MH537547 | MH542735 | MH542831 |
| *Cyphocharax sanctigabrielis* | LBP 6993 | 33399 | MH537164 | - | MH537395 | - | MH542708 | - |
| *Cyphocharax santacatarinae* | LBP 7390 | 35379 | MH537165 | - | MH537398 | MH537520 | - | - |
| *Cyphocharax santacatarinae* | LBP 7447 | 35788 | MH537166 | MH537288 | MH537399 | MH537521 | MH542710 | - |
| *Cyphocharax spilotus* | LBP 4747 | 25521 | KX087055 | KX086763 | KX086804 | KX086890 | - | - |
| *Cyphocharax spiluropsis* | LBP 1537 | 11889 | HQ171243 | KF562434 | HQ289534 | HQ288953 | HQ289150 | MH542796 |
| *Cyphocharax spiluropsis* | LBP 8876 | 44345 | MH537167 | MH537289 | MH537413 | MH537534 | MH542728 | MH542823 |
| *Cyphocharax spiluropsis* | LBP 14163 | 59220 | MH537168 | MH537290 | MH537434 | MH537554 | MH542750 | - |
| *Cyphocharax spilurus* | MHNG 2706.087 | SU07-419 157-19 | MH537169 | MH537291 | MH537364 | MH537490 | MH542686 | - |
| *Cyphocharax spilurus* | ANSP 189157 | 6877 | MH537170 | MH537292 | MH537443 | MH537562 | MH542758 | MH542843 |
| *Cyphocharax spilurus* | USNM uncat | GY11-2-74 | MH537171 | MH537293 | MH537452 | MH537571 | MH542765 | - |
| *Cyphocharax spilurus* | USNM 403679 | GY11-4-43 | MH537172 | MH537294 | MH537453 | - | MH542766 | - |
| *Cyphocharax vanderi* | LBP 3871 | 22449 | MH537173 | MH537295 | MH537382 | MH537505 | MH542700 | - |
| *Cyphocharax vanderi* | LBP 3871 | 22450 | MH537174 | MH537296 | MH537383 | MH537506 | MH542701 | - |
| *Cyphocharax voga* | LBP 3340 | 20479 | MH537175 | MH537297 | MH537378 | MH537502 | MH542713 | - |
| *Cyphocharax voga* | LBP 17002 | 68367 | MH537176 | MH537298 | MH537442 | MH537561 | MH542754 | - |
| *Potamorhina altamazonica* | LBP 2571 | 17020 | HQ171261 | - | HQ289552 | HQ288971 | HQ289168 | HQ289359 |
| *Potamorhina altamazonica* | OS 18312 | PE10-118 | MH537177 | MH537299 | MH537464 | - | MH542776 | MH542854 |
| *Potamorhina altamazonica* | OS 18312 | PE10-119 | MH537178 | MH537300 | MH537465 | - | MH542777 | - |
| *Potamorhina laticeps* | LBP 6133 | 29516 | MH537179 | MH537301 | MH537393 | MH537516 | MH542706 | MH542811 |
| *Potamorhina latior* | LBP 4252 | 22717 | MH537180 | MH537302 | MH537384 | MH537507 | MH542702 | - |
| *Potamorhina latior* | LBP 14931 | 61544 | MH537181 | MH537303 | MH537436 | MH537555 | MH542752 | MH542837 |
| *Potamorhina squamoralevis* | LBP 3768 | 22067 | MH537182 | MH537304 | MH537379 | MH537503 | MH542698 | - |
| *Potamorhina squamoralevis* | LBP 3768 | 22070 | MH537183 | MH537305 | MH537380 | MH537504 | MH542699 | - |
| *Psectrogaster amazonica* | OS 18313 | PE10-011 | MH537184 | MH537306 | MH537461 | MH537577 | MH542774 | - |
| *Psectrogaster amazonica* | OS 18313 | PE10-013 | KX087049 | KX086792 | KX086802 | - | KX086969 | - |
| *Psectrogaster ciliata* | LBP 3057 | 19164 | MH537185 | MH537307 | MH537374 | MH537498 | MH542696 | - |
| *Psectrogaster ciliata* | LBP 3057 | 19165 | MH537186 | MH537308 | MH537375 | MH537499 | MH542697 | - |
| *Psectrogaster curviventris* | LBP 657 | 8076 | MH537187 | MH537309 | MH537447 | MH537567 | MH542760 | - |
| *Psectrogaster curviventris* | LBP 3850 | 22280 | MH537188 | MH537310 | MH537381 | - | MH542714 | - |
| *Psectrogaster essequibensis* | LBP 14272 | 59531 | MH537189 | MH537313 | MH537435 | - | MH542751 | - |
| *Psectrogaster essequibensis* | USNM 401563 | GY11-1-22 | MH537190 | MH537314 | MH537450 | - | MH542763 | - |
| *Psectrogaster falcata* | LBP 13777 | 57112 | MH537191 | MH537315 | MH537427 | - | MH542747 | - |
| *Psectrogaster rhomboides* | LBP 5533 | 27204 | - | - | MH537391 | MH537514 | MH542718 | - |
| *Psectrogaster rutiloides* | OS 18315 | PE10-114 | MH537192 | MH537316 | MH537462 | - | MH542775 | - |
| *Psectrogaster rutiloides* | OS 18315 | PE10-116 | MH537193 | MH537317 | MH537463 | - | - | - |
| *Pseudocurimata boehlkei* | ROM 93679 | T13562 | MH537194 | MH537318 | MH537476 | MH537586 | MH542788 | MH542859 |
| *Pseudocurimata boehlkei* | ROM 93679 | T13563 | MH537195 | MH537319 | MH537477 | MH537587 | MH542789 | MH542860 |
| *Pseudocurimata boulengeri* | ROM 93062 | T13774 | MH537196 | MH537320 | MH537479 | MH537589 | MH542791 | MH542862 |
| *Pseudocurimata lineopunctata* | STRI-1389 | BFD01628 | MH537197 | MH537321 | MH537473 | MH537583 | MH542785 | - |
| *Pseudocurimata lineopunctata* | STRI-881 | BFD01630 | MH537198 | MH537322 | MH537475 | MH537585 | MH542787 | - |
| *Pseudocurimata troschelii* | LBP 9371 | 43968 | MH537199 | MH537323 | MH537410 | MH537531 | MH542740 | MH542820 |
| *Pseudocurimata troschelii* | Univ. Guayaquil 1542 | PSB201 | MH537200 | MH537324 | MH537469 | MH537579 | MH542781 | MH542857 |
| *Pseudocurimata troschelii* | Univ. Guayaquil 1542 | PSB202 | MH537201 | MH537325 | MH537470 | MH537580 | MH542782 | MH542858 |
| *Pseudocurimata troschelii* | ROM 93062 | T13773 | MH537202 | MH537326 | MH537478 | MH537588 | MH542790 | MH542861 |
| *Steindachnerina amazonica* | LBP 2463 | 16313 | - | MH537327 | MH537368 | MH537492 | MH542688 | MH542801 |
| *Steindachnerina argentea* | STRI-4270 | BFD01625 | MH537203 | MH537328 | MH537474 | MH537584 | MH542786 | - |
| *Steindachnerina bimaculata* | LBP 173 | 4023 | MH537204 | MH537329 | MH537405 | MH537527 | MH542724 | - |
| *Steindachnerina brevipinna* | LBP 5185 | 26336 | HQ171339 | - | HQ289628 | HQ289048 | HQ289241 | HQ289435 |
| *Steindachnerina brevipinna* | LBP 8448 | 42357 | MH537205 | MH537331 | MH537408 | MH537530 | MH542726 | MH542818 |
| *Steindachnerina brevipinna* | LBP 8573 | 43355 | MH537206 | MH537332 | MH537409 | - | MH542727 | MH542819 |
| *Steindachnerina conspersa* | LBP 14055 | 58401 | MH537207 | MH537335 | MH537432 | MH537552 | MH542748 | MH542835 |
| *Steindachnerina conspersa* | LBP 14055 | 58402 | MH537208 | MH537336 | MH537433 | MH537553 | MH542749 | MH542836 |
| *Steindachnerina dobula* | ANSP 180798 | 4123 | MH537209 | MH537337 | MH537406 | MH537528 | MH542725 | MH542816 |
| *Steindachnerina* aff. *pupula* | LBP 16874 | 49335 | MH537210 | MH537338 | MH537419 | MH537540 | MH542745 | MH542826 |
| *Steindachnerina* aff. *pupula* | LBP 16820 | 69256 | MH537211 | MH537339 | - | MH537565 | - | MH542844 |
| *Steindachnerina elegans* | LBP 8272 | 38329 | MH537212 | MH537340 | MH537401 | MH537523 | MH542712 | - |
| *Steindachnerina elegans* | LBP 8272 | 38330 | MH537213 | MH537341 | MH537402 | MH537524 | MH542720 | MH542814 |
| *Steindachnerina fasciata* | MZUSP 96802 | 6167 | MH537214 | MH537342 | MH537455 | MH537573 | MH542768 | MH542849 |
| *Steindachnerina gracilis* | LBP 8855 | 44286 | MH537215 | MH537343 | MH537411 | MH537532 | MH542741 | MH542821 |
| *Steindachnerina gracilis* | LBP 8855 | 44287 | MH537216 | MH537344 | MH537412 | MH537533 | MH542742 | MH542822 |
| *Steindachnerina guentheri* | LBP 12560 | 54206 | MH537217 | MH537345 | MH537424 | MH537545 | MH542733 | MH542829 |
| *Steindachnerina hypostoma* | ANSP 178124 | 1736 | MH537218 | MH537346 | MH537371 | MH537495 | MH542693 | MH542802 |
| *Steindachnerina hypostoma* | ANSP 178124 | 1742 | MH537219 | - | MH537372 | MH537496 | MH542694 | MH542803 |
| *Steindachnerina insculpta* | LBP 3192 | 19389 | MH537220 | MH537347 | MH537377 | MH537501 | - | MH542806 |
| *Steindachnerina insculpta* | LBP 12311 | 40021 | MH537221 | MH537348 | MH537403 | MH537525 | MH542721 | MH542815 |
| *Steindachnerina leucisca* | ANSP 178125 | 1744 | MH537222 | MH537349 | MH537373 | MH537497 | MH542695 | MH542804 |
| *Steindachnerina notonota* | LBP 2354 | 16488 | MH537223 | MH537350 | MH537369 | MH537493 | MH542691 | - |
| *Steindachnerina notonota* | LBP 2354 | 16489 | MH537224 | MH537351 | MH537370 | MH537494 | MH542692 | - |
| *Steindachnerina planiventris* | OS 18324 | PE10-144 | MH537225 | MH537354 | MH537466 | - | - | - |
| *Steindachnerina planiventris* | OS 18324 | PE10-146 | MH537226 | MH537355 | - | - | MH542779 | MH542855 |
| *Steindachnerina quasimodoi* | LBP 4125 | 23621 | MH537227 | MH537356 | MH537386 | MH537509 | - | - |
| *Steindachnerina seriata* | MZUSP 97569 | 7272 | MH537228 | MH537357 | MH537456 | MH537574 | MH542769 | MH542850 |
| *Chilodus fritillus* | AUM 51355 | T10201 | KF562391 | KF562418 | KX086863 | - | KF562495 | KX086988 |
| *Caenotropus mestomorgmatos* | ANSP 180516 | T48 | KF562384 | KF562412 | KF562442 | KX086924 | KF562490 | KX086991 |

Reference

Sabaj, M. H. (2016). Standard symbolic codes for institutional resource collections in herpetology and ichthyology: An Online Reference. Version 6.5 (16 August 2016). American Society of Ichthyologists and Herpetologists, Washington, DC. https://asih.org/sites/default/files/documents/symbolic_codes_for_collections_v6.5_2016.pdf
